# Effects of empagliflozin on right ventricular free-wall strain in patients with heart failure and reduced ejection fraction: results from the Empire HF Trial

**DOI:** 10.1093/eschf/xvaf031

**Published:** 2026-01-08

**Authors:** Massar Omar, Jesper Jensen, Mulham Ali, Peter H Frederiksen, Caroline Kistorp, Lars Videbæk, Mikael Kjær Poulsen, Christian D Tuxen, Barry A Borlaug, Sören Möller, Finn Gustafsson, Lars Køber, Morten Schou, Jacob Eifer Møller

**Affiliations:** Department of Cardiology, Odense University Hospital, J. B. Winsløws Vej 4, Odense C 5000, Denmark; Faculty of Health Sciences, University of Southern Denmark, Odense, Denmark; Steno Diabetes Centre Odense, Odense University Hospital, Odense Denmark; Department of Cardiology, Herlev and Gentofte University Hospital, Copenhagen, Denmark; Department of Cardiology, Odense University Hospital, J. B. Winsløws Vej 4, Odense C 5000, Denmark; Faculty of Health Sciences, University of Southern Denmark, Odense, Denmark; Department of Cardiology, Odense University Hospital, J. B. Winsløws Vej 4, Odense C 5000, Denmark; Faculty of Health Sciences, University of Southern Denmark, Odense, Denmark; Department of Clinical Medicine, University of Copenhagen, Copenhagen, Denmark; Department of Endocrinology, Rigshospitalet, Copenhagen, Denmark; Department of Cardiology, Odense University Hospital, J. B. Winsløws Vej 4, Odense C 5000, Denmark; Department of Cardiology, Odense University Hospital, J. B. Winsløws Vej 4, Odense C 5000, Denmark; Depatment of Cardiology, Bispebjerg and Frederiksberg University Hospital, Copenhagen, Denmark; Division of Cardiovascular Diseases, Department of Cardiovascular Medicine, Mayo Clinic, Rochester, MN, USA; Department of Clinical Research, Odense University Hospital, Odense, Denmark; Department of Clinical Medicine, University of Copenhagen, Copenhagen, Denmark; Department of Cardiology, Rigshospitalet, Copenhagen, Denmark; Department of Clinical Medicine, University of Copenhagen, Copenhagen, Denmark; Department of Cardiology, Rigshospitalet, Copenhagen, Denmark; Department of Cardiology, Herlev and Gentofte University Hospital, Copenhagen, Denmark; Department of Clinical Medicine, University of Copenhagen, Copenhagen, Denmark; Department of Cardiology, Odense University Hospital, J. B. Winsløws Vej 4, Odense C 5000, Denmark; Faculty of Health Sciences, University of Southern Denmark, Odense, Denmark; Department of Cardiology, Rigshospitalet, Copenhagen, Denmark

**Keywords:** SGLT2i, Empagliflozin, heart failure with reduced ejection fraction, right ventricular function, right ventricular free wall strain

## Abstract

**Introduction:**

To investigate the effect of empagliflozin on right ventricular (RV) function in patients with heart failure with reduced ejection fraction (HFrEF). Sodium-glucose cotransporter-2 (SGLT2) inhibitors improve outcomes and reverse left ventricular (LV) remodelling in HFrEF. The impact on RV function remains uncertain.

**Methods:**

The Empire HF trial was an investigator-initiated, double-blind, randomized, placebo-controlled trial of 190 participants with a left ventricular ejection fraction (LVEF) of 40% or lower, with New York Heart Association (NYHA) Class I−III symptoms. Participants were randomized to receive either empagliflozin (at a dose of 10 mg once daily) or placebo, on top of recommended therapy for 12 weeks. The primary endpoint of this *exploratory substudy* was changes in RV free wall strain (RVFWS) across the whole cohort. RVFWS was also stratified into tertiles based on baseline RVFWS. Secondary endpoints included changes in tricuspid annular plane systolic excursion (TAPSE) and RV S’ velocity.

**Results:**

Between June 2017 and September 2019, a total of 190 patients were enrolled, of whom 160 were included in this substudy. Baseline characteristics were balanced between the groups (mean age of 64 ± 11 years, 86% male, mean LVEF: 29 ± 8%, 79% in NYHA Class II, 83 patients (52%) had an implanted cardiac device, and tricuspid valve regurgitation was absent/trace in 136 (85%) and mild in 24 (15%)). A high proportion of the population were on optimal medical treatment for HFrEF, and device therapy remained unchanged during follow-up. The overall mean RVFWS was −16.7 ± 6.1%, with the empagliflozin group at −16.4 ± 6.2% and the placebo group at −16.9 ± 5.9%. No differences were observed in RVFWS between the groups. When stratified by baseline RVFWS into tertiles, patients in the lowest tertile demonstrated a significant improvement with empagliflozin (treatment effect: −2.9% [95% CI: −5.0 to −0.3]; *P* = .027). This finding was independent of LVEF, plasma volume, and weight loss, and remained unchanged after additional adjustment for LV remodelling and the presence of resynchronization therapy. No significant treatment effect was observed in the middle or highest tertiles of RVFWS, nor in the overall or lowest tertiles of TAPSE or RV S’.

**Conclusion:**

This exploratory substudy of the Empire HF, treatment with empagliflozin exerted no overall effect on RV function in stable HFrEF patients, but significantly improved RVFWS in patients in the lowest tertile of RVFWS after 12 weeks of treatment.

**Trial Registration:**

ClinicalTrials.gov, Unique Identifier: NCT03198585

## Graphical Abstract

**Graphical Abstract:**
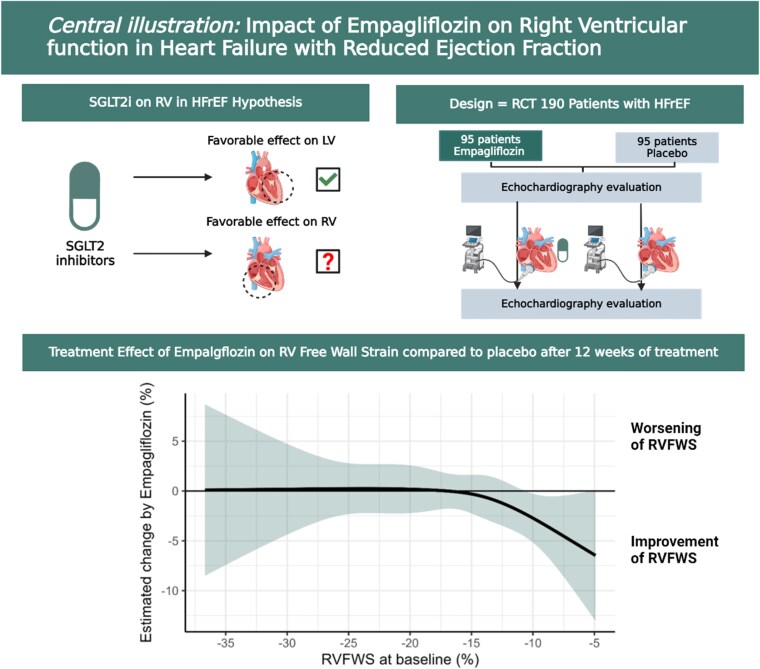
Illustrative overview of aim, study design and results of the study. Abbreviations: SGLT2i, sodium-glucosetransporter-2 inhibitor; RV, right ventricle; HFrEF, heart failure with reduced ejection fraction; LV, left ventricle; RCT, randomized clinical trial; RVFWS, right ventricular free wall strain

## Introduction

Dysfunction of the right ventricle (RV) is common in heart failure (HF) with reduced ejection fraction (HFrEF), occurring in 35%–50% of patients.^1^ RV dysfunction is associated with progression and prognosis in HF patients,^2^ with earlier studies showing that each 1% worsening in RV free wall strain (RVFWS) was independently associated with a 5% increased risk of all-cause mortality.^3^

Sodium-glucose transporter-2 (SGLT2) inhibitors have emerged as a potent HFrEF therapy, improving clinical outcomes and quality of life in patients with HF irrespective of left ventricular ejection fraction (LVEF).^4–7^ The exact mechanisms underlying the beneficial effects remain partially elucidated. The benefits may be related to improved hemodynamics including, but not restricted to, reduced preload, reverse left ventricular (LV) remodelling, and metabolic changes.^8–10^

Knowledge regarding the effect of SGLT2 inhibitors on RV function remains remarkably limited. A small, single-blinded study conducted in 36 patients with HFrEF, along with two observational studies, found that empagliflozin significantly improved the systolic and global RV function assessed by RVFWS and tricuspid annular plane systolic excursion (TAPSE) after 12 and 24 weeks of treatment, respectively.^11–13^

However, observational studies are subject to bias and cannot provide definite proof of the effect of an intervention. To distinguish treatment effects from the natural progression of the disease, a control group is required.

Therefore, the aim of this exploratory substudy analysis of the Empagliflozin in Heart Failure Patients with Reduced Ejection Fraction (Empire HF) trial was to investigate the impact of empagliflozin on RV function in 190 patients with HFrEF after 12 weeks of treatment compared to placebo.^14^

## Methods

### Study design and ethics

This is an exploratory substudy analysis from the Empire HF trial, which has been previously published.^14,15^ The trial was an investigator-initiated, multicentre, randomized, double-blind, placebo-controlled clinical trial, randomly assigning 190 patients with HFrEF (1:1) to empagliflozin 10 mg once daily or a matching placebo for 12 weeks. At baseline, patients underwent a comprehensive clinical examination, transthoracic echocardiography, and blood tests, which were repeated at 12-week follow-up.

The trial was conducted in accordance with Good Clinical Practice and the Declaration of Helsinki. All participants signed informed consent before inclusion. The Empire HF trial is registered in ClinicalTrials.gov, NCT03198585.

### Study participants

Stable HFrEF patients aged ≥18 years, on optimal HF therapy in accordance with European and national guidelines,^16^ with a LVEF ≤40%, and with a New York Heart Association (NYHA) Class I–III, clinically euvolemic, and without recent (<30 days) heart failure hospitalization or major medication changes, were eligible for this study. Patients with uncorrected severe valvular disease or other comorbidities interfering with participation were excluded. The full list of inclusion and exclusion criteria is presented in the Appendix (Supplementary Appendix, section C).

### Echocardiography

Transthoracic echocardiography was performed on a Vivid e9 ultrasound system (General Electric, Horten, Norway) and stored digitally for offline analysis using Echopac (version 203, General Electric). Echocardiographic measurements were not pre-specified in the statistical analysis plan and were decided after the end of the trial,^15^ but before un-blinding of the study. Analyses were performed in the intention-to-treat population, including all randomized patients with complete data, analysed in a random order and blinded to treatment allocation.

The function of the RV was assessed by measuring RVFWS, TAPSE, and RV S’ wave in accordance with current guidelines.^17^ TAPSE was measured in the Apical 4 Chamber (A4C)-view using the M-mode cursor over the lateral tricuspid annulus and measuring a straight line from top to bottom of the wave, which calculates the systolic displacement of the tricuspid annulus, recorded in cm. RV S’ wave was measured using tissue Doppler imaging (TDI) from the A4C view by placing the Doppler cursor on the lateral tricuspid annulus to record the peak systolic velocity (S’ wave) in cm/s, representing RV longitudinal systolic function. The maximal value was chosen for analysis. RVFWS was measured using Q-analysis of the Echopac software from the apical four-chamber view. The region of interest was set to cover the tricuspid insertion, from the lateral RV free wall to the RV apex. RVFWS is calculated as the percentage of systolic shortening of the RV free wall from base to apex^17^ and is reported as a negative value.

LV volumes and LVEF were assessed using the biplane method of disks (Simpsons´s Biplane). A detailed description of the echocardiography protocol is provided in the Appendix (Supplementary Appendix, section D).

### Efficacy measures

The primary efficacy measure in the exploratory substudy was the between-group difference in the change in RVFWS from baseline to 12-week follow-up. Secondary exploratory measures included the between-group differences in the changes of TAPSE and RV S’ velocity.

### Statistical analysis

No specific sample size estimation was performed for this exploratory substudy analysis, and the study population size was the sample size of the main Empire HF trial.^15^ The primary statistical analysis was conducted in the intention-to-treat population, including all patients with available RVFWS measurements, with no imputation of missing echocardiographic data. Sensitivity analyses were performed using available data on TAPSE and RV S’, and the results remained consistent with the intention-to-treat findings (Supplementary Table S3).

Baseline characteristics and echocardiographic measurements were reported as means and standard deviations (SD) for normally distributed variables, numbers (%) for categorical variables, and medians with interquartile ranges (IQR) for non-normally distributed variables.

Patients were stratified into subgroups based on tertiles of baseline values of RVFWS. The subgroups values were derived from the distribution of RVFWS at baseline in the study population, as the existing literature does not provide a consensus on the appropriate diagnostic and prognostic cut-off values for patients with HFrEF.^18^ Furthermore, to visualize and delineate the associations of treatment effect on RVFWS, we further tested the non-linear relationship between empagliflozin vs placebo on RVFWS (as a continuous variable) using restricted cubic spline curves.

The between-group difference was based on a per-protocol analysis, adjusted for age, sex, diabetes, atrial fibrillation, and coronary artery bypass grafting (CABG).

Subgroup analyses were interpreted with mean change and 95% confidence intervals (CI) as the change between the groups by the specified subgroups. The *P*-value denotes the two-way interaction between subgroups.

All statistical tests were performed for a two-sided 0.05 alpha-level of significance and reported with the corresponding 95% CI. Statistical analyses were conducted using STATA statistical software, version 17 (Stata Crop, College Station, Texas, USA).

## Results

### Baseline characteristics

Of 697 participants screened between 29 June 2017 and 10 September 2019, 95 participants were randomly assigned to empagliflozin and 95 assigned to placebo. Both baseline and 12-week follow-up echocardiographic measurements of RVFWS were available for 82 patients in the empagliflozin group and 78 in the placebo group, and these patients were included in the primary intention-to-treat analysis (Supplementary Fig. S1). The groups did not differ with respect to baseline characteristics (*Table 1*). The mean age was 64 years, 138 (86%) were male, 126 (79%) reported a NYHA Class II, 83 patients (52%) had an implanted cardiac device, and a high proportion were on optimal medical treatment for HFrEF. There were no clinically meaningful between-group differences in concomitant HF therapy, including diuretic dosages at baseline (*Table 1*), and no significant changes between groups during the study period. Tricuspid valve regurgitation was absent or trace in 136 (85%) patients and mild in 24 (15%), with no between-group differences. No device implantation or device therapy occurred during follow-up. All patients, except one, had medication compliance greater than 90% (Supplementary Fig. S1).

**Table 1:** Baseline characteristics

Characteristic	Empagliflozin *n* = 82	Placebo *n* = 78
**Age, mean (SD), years**	64 (10)	63 (12)
**Male, no. (%)**	70 (85)	68 (87)
**Body mass index, median (IQR), kg/m^2^**	29 (26–32)	28 (26–32)
**Heart failure characteristics**		
Duration of heart failure, median (IQR), months	36 (12–69)	27 (12–73)
Ischaemic heart failure aetiology, no. (%)	48 (51)	49 (52)
NYHA Class, no. (%)		
*I*	5 (6)	7 (9)
*II*	62 (75)	64 (82)
*III*	15 (18)	7 (9)
Device type, no. (%)		
Cardiac resynchronization therapy with ICD	5 (11)	3 (7)
Cardiac resynchronization therapy without ICD	9 (20)	12 (29)
ICD only	30 (68)	24 (59)
**Comorbidities, mean (SD), no. (%)**		
Type 2 diabetes	9 (11)	10 (13)
Hypertension	32 (39)	31 (40)
Atrial fibrillation	26 (32)	25 (32)
**Medications, mean (SD), no. (%)**		
*ACE inhibitors/ARBs*	54 (66)	56 (72)
*Sacubitril–valsartan*	24 (29)	19 (24)
β-blockers	77 (93)	73 (94)
Mineralocorticoid-receptor antagonist	52 (63)	51 (65)
Diuretics^a^	53 (65)	51 (65)
**Laboratory variables**		
NT-proBNP, median (IQR), ng/L	522 (303–944)	623 (262–1080)
Estimated glomerular filtration rate, median (IQR), mL/min/1.73m^2^	73 (57–89)	74 (60–90)
Haematocrit, mean (SD), %	42 (4)	41 (5)
**Echocardiography**		
***Left Ventricle***		
LV End-diastolic volume, mean (SD), mL	170 (81)	165 (60)
LV End-systolic volume, mean (SD), mL	115 (68)	108 (48)
LV Mass Index, mean (SD), g/m^2^	130 (66)	129 (45)
LVEF, mean (SD), %	35 (9)	36 (9)
LV Global longitudinal strain, mean (SD), %	−11.4 (3.7)	−11.4 (3.6)
LA Volume index, mean (SD), mL/m^2^	40 (18)	37 (13)
***Right Ventricle***		
RV Free wall strain, mean (SD), %	−16.4 (6.3)	−16.9 (5.9)
TAPSE, mean (SD), cm	2.1 (0.6)	2.1 (0.6)
RV S’, mean (SD), m/s	0.10 (0.03)	0.11 (0.03)
TR_max_PG, mean (SD), mmHg	21 (13)	21 (9)

Baseline characteristics of the empagliflozin and placebo group in patients with heart failure and reduced ejection fraction with available RVFWS measurements.

NYHA, New York Heart Association; ACE inhibitors, angiotensin-converting enzyme inhibitors; ARB, angiotensin-II receptor blockers; NT-proBNP, N-terminal pro-B-type natriuretic peptides; LV, left ventricle, LA, left atrium; RV, right ventricle; TAPSE, tricuspid annular plane systolic excursion; TR_max_PG, tricuspid regurgitation peak gradient; RV S’, right ventricular systolic velocity. SD, standard deviation; IQR, interquartile range (25% and 75% percentiles).

^a^Diuretics includes loop diuretics or thiazide.

Baseline characteristics of the tertiles of RVFWS are presented in *Table 2*. The baseline means of RVFWS were −23.3% (SD, 4.4), −16.0% (SD, 1.4), and −10.5% (SD, 2.6) for the upper, middle, and lower tertiles, respectively. Patients in the lower tertile of RVFWS had a longer duration of HFrEF, lower LVEF, higher body weight, and a greater prevalence of diabetes and atrial fibrillation, with a higher proportion (77%) of them on diuretics, compared to 65% in the entire cohort. Additionally, they had the highest LV end-diastolic and end-systolic volumes, LV mass index, left atrial volume index, RV S’, and the lowest LVEF compared to middle and upper tertile groups. At baseline, there were no significant differences between subgroups in the use of guideline-directed medical therapy (GDMT), including diuretics or in estimated glomerular filtration rate (*Table 2*).

**Table 2:** Baseline characteristics of the population and subgroups

Characteristic	Total	Upper RVFWS tertile	Middle RVFWS tertile	Lower RVFWS tertile	*P*-value
	*n* = 160	*n* = 54	*n* = 53	*n* = 53	
**Age, mean (SD), years**	64 (11)	68 (9)	61 (14)	61 (10)	.001
**Male, no. (%)**	138 (86)	43 (80)	48 (91)	47 (89)	.21
**Body mass index, median (IQR)**	28.4 (25.9–32.0)	26.9 (24.7–31.2)	28.3 (26.0–32.4)	29.6 (26.9–32.9)	.01
**Heart failure characteristics**					
Duration of heart failure, median (IQR), months	28 (12–71)	18 (11–45)	21 (9–59)	58 (18–100)	<.001
Ischaemic heart failure cause, no. (%)	78 (49)	26 (48)	30 (57)	22 (42)	.30
NYHA Class, no. (%)					.30
*I*	12 (8)	6 (11)	2 (4)	4 (8)	
*II*	126 (79)	44 (81)	43 (81)	39 (74)	
*III*	22 (14)	4 (7)	8 (15)	10 (19)	
Device type, no. (%)					.53
Cardiac resynchronization therapy with ICD	8 (9)	4 (17)	2 (6)	2 (6)	
Cardiac resynchronization therapy without ICD	21 (25)	3 (13)	9 (29)	9 (29)	
ICD Only	54 (64)	15 (65)	19 (61)	20 (65)	
**Comorbidities, mean (SD), no. (%)**					
Type 2 diabetes	19 (12)	4 (7)	3 (6)	12 (23)	.01
Hypertension	63 (39)	22 (41)	25 (47)	16 (30)	.20
Atrial fibrillation	51 (32)	13 (24)	15 (28)	23 (43)	.08
**Medications, mean (SD), no. (%)**					
ACE inhibitors/ARBs	110 (69)	37 (69)	35 (66)	38 (72)	.82
Sacubitril–valsartan	43 (27)	14 (26)	15 (28)	14 (26)	.96
β-Blockers	150 (94)	53 (98)	48 (91)	49 (92)	.24
Mineralocorticoid-receptor antagonist	103 (64)	35 (65)	39 (74)	29 (55)	.13
Diuretics	104 (65)	32 (59)	31 (58)	41 (77)	.07
**Laboratory variables**					
NT-proBNP, median (IQR), ng/L	565 (294–1030)	476 (246–876)	544 (304–809)	854 (303–1230)	.22
Estimated glomerular filtration rate, median (IQR), mL/min/1.73m^2^	73.0 (58.5–89.5)	73.0 (59.0–89.0)	72.0 (59.0–90.0)	73.0 (58.0–84.0)	.71
Haematocrit, mean (SD), %	41 (4)	40 (4)	42 (5)	42 (4)	.006
**Echocardiography**					
***Left Ventricle***					
LV End-diastolic volume, mean (SD), mL	168 (72)	135 (58)	183 (61)	185 (83)	<.001
LV End-systolic volume, mean (SD), mL	112 (59)	81 (45)	123 (50)	13 (70)	<.001
LV Mass index, mean (SD), g/m^2^	130 (57)	107 (29)	128 (44)	155 (76)	<.001
LVEF, mean (SD), %	35(9)	42 (10)	34 (7)	31 (7)	<.001
LV Global longitudinal strain, mean (SD)	11.4 (3.6)	14.0 (3.3)	11.2 (3.1)	9.0 (2.7)	<.001
LA Volume index, mean (SD), mL/m^2^	39 (16)	35 (12)	37 (11)	44 (22)	.008
***Right Ventricle***					
RV Free wall strain, mean (SD), %	−16.7 (6.1)	−23.3 (4.4)	−16.0 (1.4)	−10.5 (2.6)	By design
TAPSE, mean (SD), cm	2.1 (0.6)	2.4 (0.5)	2.1 (0.5)	1.8 (0.5)	<.001
RV S’, mean (SD), m/s	0.11 (0.28)	0.12 (0.25)	0.12 (0.29)	0.10 (0.27)	<.001
TR_max_PG, mean (SD), mmHg	21 (11)	21 (9)	20 (10)	22 (14)	.46

Baseline characteristics of the total population and tertiles based on RVFWS at baseline in patients with heart failure and reduced ejection fraction. *P*-values reflect between-group differences, tested by Pearson’s chi-square test if not listed. Bold values indicate significance.

NYHA, New York Heart Association; ACE inhibitors, angiotensin-converting enzyme inhibitors; ARB, angiotensin-II receptor blockers; NT-proBNP, N-terminal pro-B-type natriuretic peptides; LV, left ventricle, LA, left atrium; RV, right ventricle; TAPSE, tricuspid annular plane systolic excursion; TRmaxPG, tricuspid regurgitation peak gradient; RV S', right ventricular systolic velocity. SD, standard deviation; IQR, interquartile range (25% and 75% percentiles).

### Primary efficacy measure

**Figure 1:**
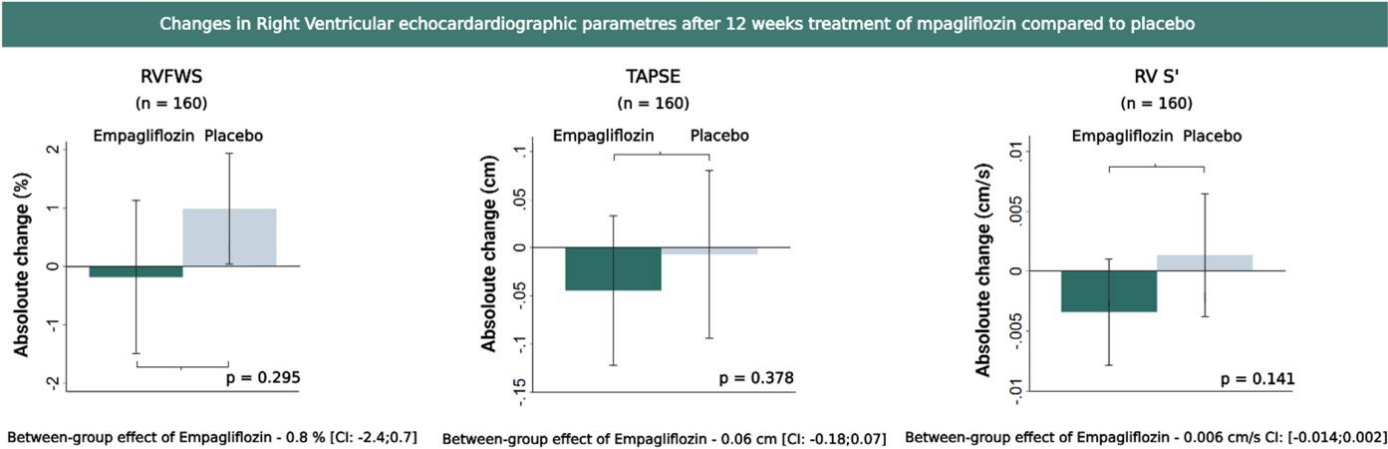
Effects on right ventricular parameters in the total population. The bars illustrate the mean change in the Empagliflozin and placebo group from baseline to follow-up after 12 weeks of treatment. Treatment effect of Empagliflozin between groups is listed with confidence intervals and *P*-value. Empagliflozin exerted no effect on RVFWS, TAPSE, and RV S’ after 12 weeks of treatment with Empagliflozin compared to placebo. RVFWS, right ventricular free wall strain; TAPSE, tricuspid annular plane systolic excursion; RV S’, right ventricular systolic velocity

At baseline, participants had an overall RVFWS mean of −16.7% (SD, 6.1), with the empagliflozin group at −16.4% (SD, 6.2) and the placebo group at −16.9% (SD, 5.9). The overall between-group treatment effect after 12 weeks was not statistically significant [adjusted treatment effect: −0.8% (95% CI: −2.4 to 0.7); *P*  *=* .30] (*Table 3*, *Figure 1*).

**Table 3:** Efficacy measures

	Empagliflozin						Placebo						Between group		
	Baseline		Follow-up		Mean change (SD)		Baseline		Follow-up		Mean change (SD)		Adjusted treatment effect [95% CI]		*P*-value
**Cardiac measurements, right ventricle**															
RVFWS, mean [SD], %	−16.4	[6.2]	−16.0	[5.8]	−0.2	[5.5]	−16.9	[5.9]	−16.0	[5.4]	1.0	[4.0]	−2.4	[−2.4 to 0.7]	0.295
Lower RVFWS tertile, mean [SD], %	−9.7	[2.4]	−14.1	[4.9]	−4.2	[5.0]	−11.3	[2.6]	−12.7	[5.1]	−0.9	[4.4]	−2.9	[−5.0 to −0.3]	0.027
Middle RVFWS tertile, mean [SD], %	−15.9	[1.6]	−14.8	[5.1]	1.1	[4.9]	−16.1	[1.2]	−14.7	[2.67]	1.4	[2.4]	−0.3	[−2.4 to 1.8]	0.786
Upper RVFWS tertile, mean [SD], %	−23.4	[3.9]	−20.9	[4.9]	2.7	[4.3]	−23.2	[4.9]	−20.5	[5.12]	2.2	[4.5]	0.2	[−2.4 to 2.6]	0.943
TAPSE, mean [SD], cm	2.1	[0.6]	2.0	[0.6]	−0.1	[0.4]	2.1	[0.6]	2.1	[0.6]	0.0	[0.4]	−0.06	[−0.18 to 0.07]	0.378
RV S’, mean [SD], m/s	0.11	[0.03]	0.10	[0.03]	−0.01	[0.02]	0.11	[0.03]	0.11	[0.03]	0.01	[0.03]	−0.006	[−0.0136 to 0.0019]	0.141
TR _max_PG, mean[SD], mmHg	21	[12]	20	[10]	−0.6	[11]	21	[9]	21	[9]	0.3	[9.0]	−0.7	[−3.9 to 1.8]	0.646
**Cardiac measurements, left ventricle**															
LVEF, mean [SD], %	35	[9.2]	37	[10.9]	2.4	[7.5]	36	[9.3]	38	[9.0]	1.0	[8.4]	1.2	[−1.2 to 3.6]	0.315
LV Mass index, mean [SD], g/m^2^	41	[20]	40	[17]	−1.1	[8]	36	[13]	37	[13]	1.4	[8]	−2.5	[−4.4 to −0.8]	0.043
LV End-diastolic volume, mean (SD), mL	167	[79]	154	[57]	−8.9	[30]	159	[60]	160	[57]	2.8	[39.5]	−12	[−22.4 to −1.2]	0.029
LV End-systolic volume, mean (SD), mL	112	[66]	100	[50]	−8.5	[26]	104	[48]	102	[44]	0.2	[31.2]	−9	[−17.5 to −0.2]	0.046
**Blood Pressure**															
Systolic, mean (SD), mmHg	119	[18]	115	[14]	−4.4	[15]	121	[16]	121	[14]	0.2	[13]	−5	[−8.6 to −0.6]	0.023
Diastolic, mean (SD), mmHg	72	[11]	71	[10]	−1.0	[10]	74	[12]	72	[12]	−1.3	[9]	0.3	[−2.5 to 3.0]	0.838
**Blood tests**															
NT-proBNP, median (IQR), ng/L	582		478		−47 [660]		605		520		3 [675]		−50	[−240 to 141]	0.610
	[303–1020]		[278–968]				[309–1080]		[262–1085]						
Haematocrit, mean [SD], %	42	[4]	44	[4]	2.1	[2.4]	41	[4]	41	[4]	−0.1	[2.3]	2.1	[1.47 to 2.81]	<0.001
Plasma volume, mean [SD], mL	3040	[409]	2898	[383]	−135	[135]	3181	[460]	3187	[424]	9	[144]	−144	[−184 to −104]	<0.001
**Metabolic**															
Weight, mean [SD], kg	90.8	[16.6]	89.4	[16.1]	−1.18	[1.8]	94.2	[18.4]	94.2	[17.6]	0.16	[2.64]	−1.3	[−2.0 to −0.7]	<0.001
BMI, mean [SD], kg/m^2^	29.3	[4.5]	28.9	[4.3]	−0.4	[0.6]	29.8	[5.4]	29.7	[5.2]	0.1	[0.8]	−0.4	[−0.6 to −0.2]	<0.001

Baseline and follow-up values for different efficacy measures in HFrEF patients receiving either Empagliflozin (10 mg daily) or placebo for 12 weeks. Adjusted treatment effect of empagliflozin compared to placebo with their respective *P*-value. The population with available RVFWS has been sub-grouped in tertiles and is presented by these in the table.

RVFWS, Right ventricular free wall strain; TAPSE, tricuspid annular plane systolic excursion; RV S’, right ventricular systolic velocity; TR maxPG, Tricuspid Regurgitation Peak Gradient; LVEF, left ventricular ejection fraction; LV, left ventricle; NT-proBNP, N-terminal pro-B-type natriuretic peptides; BMI, body mass index; SD, standard deviation; IQR, interquartile range (25% and 75% percentiles).

Baseline means of the RVFWS tertiles were as follows: for the empagliflozin group, the upper tertile was −23.4% (SD, 3.9), middle tertile −15.9% (SD, 1.6), and lower tertile −9.7% (SD, 2.4); for the placebo group, −23.2% (SD, 4.9), −16.1% (SD, 1.2), and −11.3% (SD, 2.6), respectively (*Table 3*).

**Figure 2:**
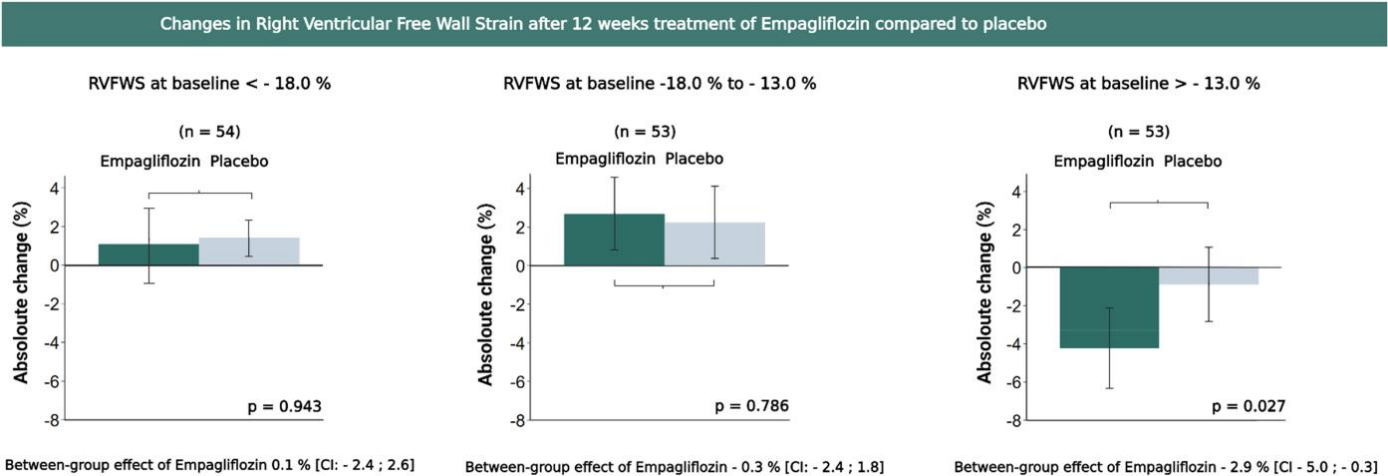
Effect on right ventricular free wall strain across subgroups. The bars represent the mean change from baseline to follow-up in RVFWS after 12 weeks of treatment with Empagliflozin compared to placebo in the subgroups stratified into tertiles based on RVFWS values at baseline. Treatment effect of Empagliflozin between groups is listed with confidence intervals and *P*-value. No significant differences were found in the upper and middle tertile; however, empagliflozin significantly improved RVFWS in the lowest tertile, compared to placebo. Abbreviations: RVFWS, Right Ventricular Free Wall Strain

In patients with the lowest RVFWS, empagliflozin treatment was associated with a significant improvement in RVFWS from baseline to follow-up compared to the placebo group (adjusted treatment effect: −2.9% [95% CI: -5.0 to −0.3]; *P* = .03) (*Table 3*, *Figure 2*). This finding remained unchanged after additional adjustment for the presence of resynchronization. There were no differences between empagliflozin and placebo in the upper or middle RVFWS tertiles (*Table 2*, *Figure 1*).

**Figure 3:**
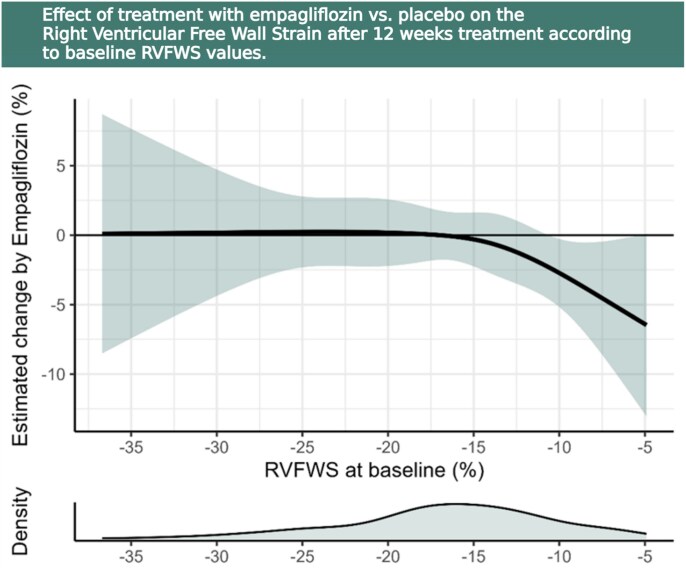
Estimated change in RVFWS by 12 weeks of treatment with Empagliflozin relatively to RVFWS at baseline. Estimated effect of Empagliflozin compared to placebo after 12 weeks of treatment based on RVFWS at baseline. Density depicts the distribution of RVFWS values at baseline. Modelled non-linearly using restricted cubic splines. Empagliflozin does not affect RVFWS after 12 weeks of treatment compared to placebo in those with RVFWS at baseline <−15%, but improves RVFWS in those with >−15%. RVFWS, right ventricular free wall strain

As depicted in *Figure 3*, patients with the lowest baseline RVFWS values experienced the greatest improvement with empagliflozin treatment, corroborating the tertile stratification analysis.

The improvement in RVFWS was not associated with weight loss, plasma volume, or haematocrit (*P* > .05). No significant correlations were observed between RVFWS and LV remodelling parameters (LVESV, LVEDV, LVM, LAVI, or systolic blood pressure (SBP)) at follow-up (all *P* > .5) (Supplementary Table S2). Finally, changes in RVFWS from baseline to 12 weeks were not significantly associated with the changes in LV remodelling parameters (LVESV, LVEDV, LVM, LAVI, or SBP) in either treatment group, with no significant between-group differences observed (all *P* > .5) (Supplementary Table S2*A*).

### Secondary efficacy measures and correlations

Compared to placebo, empagliflozin did not have a significant impact on TAPSE [adjusted treatment effect: −0.06 cm (95% CI: −0.18 to 0.07); *P* = .78] or RV S’ [adjusted treatment effect: −0.006 cm/s (95%: −0.014 to 0.002); *P* = .14], on the overall population (*Table 3*, *Figure 1*). These findings were consistent when patients were stratified into baseline tertiles of TASPE and RV S’ (Supplementary Table S3).

## Discussion

To our knowledge, this is the first study to report the effect of SGLT2 inhibition on RV function in a double-blind, randomized, controlled trial including a sizable cohort of stable, euvolemic patients with HFrEF. The study shows that empagliflozin did not yield a discernible effect on RV function in the overall study population compared to placebo after 12 weeks of treatment, but significantly improved RVFWS in patients with the lowest RVFWS at baseline. No significant changes were seen in TAPSE or the RV S’ velocity.

A smaller single-blinded, prospective study of 36 patients with HFrEF showed that empagliflozin significantly improved RVFWS after 12 weeks (baseline RVFWS −16.4%).^11^ Additionally, two observational studies demonstrated improvements in RV systolic function and pulmonary arterial stiffness, with correlations to improved NYHA classification and quality of life.^12,13^

In contrast, despite having a similar baseline RVFWS, our substudy did not find a significant treatment effect in the overall cohort. This discrepancy may be attributed to differences in patient populations. The participants in our study were well-managed, euvolemic, and exhibited only mild symptoms, which may explain why significant improvements in RVFWS were observed exclusively in individuals within the lowest baseline RVFWS tertile. This differs from the three previous studies,^11–13^ where patients were more decompensated, had larger cardiac chambers, and exhibited greater RV impairment. Conversely, stable HFrEF patients with absent or mild RV dysfunction may not derive a detectable benefit from empagliflozin on RV function, underscoring the potential for patient selection to influence treatment responses. However, the precise mechanisms behind this benefit remain unclear.

We selected RVFWS as the primary efficacy measure because data have shown that it is a better predictor of outcomes than RV global longitudinal strain, purportedly due to being less influenced by LV longitudinal dysfunction.^19^ Additionally, RVFWS serves not only as a prognostic parameter but also as a marker capable of detecting subtle deterioration in RV systolic function, even when TAPSE and RV S’ wave remain preserved, as observed in our cohort.^20–22^

It has been proposed that one of the main mechanisms by which SGLT2 inhibitors exert their cardioprotective effects is a reduction in preload, primarily due to the diuretic and natriuretic effects.^23^ We observed an increase in haematocrit and a decrease in LV end-diastolic volume from baseline to 12 weeks with empagliflozin compared with placebo, consistent with existing studies in the literature.^4,6,8,9^ However, the change in RVFWS observed in the subgroup with the lowest baseline RVWFS in the current study was not linked to alterations in haematocrit, plasma volume, weight loss, LV structure and function, and as such, the observed effects are likely not explained by the diuretic effect of empagliflozin.^24^

Right ventricular function is highly sensitive to loading conditions, particularly afterload.^25^ Compared with the left ventricle, the RV operates under a substantially wider range of systolic pressures (≈15 mmHg under normal conditions up to 150 mmHg in pulmonary hypertension), and for a given change in systolic pressure, RV systolic volumes change more dramatically, roughly three times more than LV volumes.^26^ Given the physiology of the RV, not surprisingly, RV dysfunction is influenced by both pressure and volume changes. Whether the improvement in RVFWS is due to (i) enhanced contractile function of the RV myocardium; (ii) a decrease in afterload secondary to diuretic therapy; or (iii) reductions in systemic or pulmonary artery pressures cannot be definitively determined. Changes in RVFWS from baseline to 12 weeks were not significantly associated with the changes in LV remodelling parameters, and this finding remained unchanged after additional adjustment for the presence of resynchronization therapy, supporting a beneficial effect beyond the ventricular interdependence of LV improvement. We did not measure or estimate pulmonary artery pressures; however, a subset of participants (*n* = 70) underwent right-heart catheterization, which revealed no changes in pulmonary artery pressure or pulmonary vasculature during rest or exercise, while a modest decrease in LV filling pressure was observed in the empagliflozin group compared to placebo.^23^

Taken together, the mechanisms underlying RVFWS improvement are likely multifactorial. The pronounced changes in fluid status remain among the most notable effects of SGLT2 inhibitors in short-term trials, and while enhanced diuretic efficiency may have contributed, additional mechanisms cannot be excluded. Importantly, this study is exploratory, which should be taken into account when interpreting the cardiac reverse remodelling results.

The significant improvement in RVFWS among patients in the lowest tertile raises the possibility of regression towards the mean, as these individuals had more impaired RV function at baseline. However, the absence of similar improvements in the middle or highest tertiles, or in other indices of RV function such as TAPSE or RV S’, supports the notion that the observed effect may reflect a true, treatment-specific benefit of empagliflozin in patients with more advanced RV dysfunction.

This cohort was predominantly stable, well-treated, euvolemic NYHA II patients, and mostly male. These characteristics limit generalizability to more symptomatic, congested, or female HFrEF populations. The predominance of male participants further constrains sex-specific extrapolation, as women with HFrEF may exhibit distinct ventricular-vascular coupling, smaller chamber sizes, and different remodelling patterns, which could influence baseline RV function and treatment response.

While this was a stable cohort, the beneficial effect of SGLT2 inhibition was only observed in the lowest tertile of RVFWS, representing a sicker subset with more advanced RV dysfunction and higher diuretic use. As RV function is highly sensitive to acute changes in preload and afterload, in more congested or decompensated patients, the primary effect of SGLT2 inhibition would likely be diuresis and reduction of filling pressures, which could immediately enhance RV contractility via the Frank–Starling mechanism. However, the improvement in RVFWS observed in this study also suggests a potential remodelling effect, indicating that SGLT2 inhibition may confer intrinsic improvements in RV myocardial function over time, independent of acute haemodynamic changes.

Given the short follow-up, absence of fractional area change (FAC) and Cardiovascular Magnetic Resonance data, and the potential for type I error due to multiple testing, the results should be interpreted with caution. Future studies dedicated to RV function are warranted to confirm and clarify the mechanisms and clinical implications of targeting RV function with SGLT2 inhibitors.

## Limitations

The results of this exploratory substudy should be regarded as hypothesis-generating. While the widely used parameter in the clinical setting is TAPSE, RVFWS was chosen as the primary measurement in our study, as RVFWS has been demonstrated to be superior and a more reliable echocardiographic measure compared to TAPSE,^3,27^ and to correlate better with RV ejection fraction in cardiac magnetic resonance.^28^ While prognosis and remodelling are not interchangeable, and a more comprehensive assessment including FAC would have strengthened our analysis, the absence of FAC measurements represents a limitation. However, FAC was not assessed due to potential methodological pitfalls, including limited acoustic windows, dense right ventricular trabeculations, frequent foreshortening, and incomplete RV visualization. We used echocardiography, which is less accurate and reproducible than cardiac magnetic resonance but remains the most widely utilized method. Despite its limitations, our study showed a consistent effect on RV function in the subgroup analysis.

The HFrEF patients included in this study were relatively young, euvolemic, well-compensated, and the majority were in the NYHA Class II. The applicability of our findings to HFrEF patients with more advanced disease remains uncertain and requires further investigation. Moreover, considering the short treatment duration of 12 weeks, it remains unclear whether a more pronounced effect of empagliflozin would have been observed over a longer treatment period.

## Conclusions

This exploratory substudy demonstrated that empagliflozin had no significant effect on RV function in the overall cohort of HFrEF patients but significantly improved RVFWS in the lowest tertile, with no changes in the other tertiles after 12 weeks of treatment. These benefits were independent of loading conditions, weight, resynchronization therapy, and LVEF improvement. Further studies are warranted to determine the clinical significance in HFrEF patients.

*Clinical perspective*: In euvolemic, stable patients with chronic HFrEF, empagliflozin exerted no overall effect on RV function, but significantly improved RVFWS in patients with the lowest tertile of RVFWS after 12 weeks of treatment. The benefits were unrelated to loading conditions, weight, or improvement of LVEF.

*Translational outlook*: Empagliflozin treatment may affect RV function in individuals with the lowest RVFWS values, potentially contributing to the cardioprotective properties of SGLT2 inhibitors. Further research is needed to clarify the mechanisms by which SGLT2 inhibitors improve RV function in patients with HFrEF.

## Supplementary Material

xvaf031_Supplementary_Data

## Declarations

### Data Availability

No data were generated or analysed for this manuscript.

### Pre-registered Clinical Trial Number

None supplied.
