# Supplementary material for: Effects of empagliflozin on right ventricular free-wall strain in patients with heart failure and reduced ejection fraction: results from the Empire HF Trial
Source: ESC Heart Fail. 2026 Jan 8;13(3):xvaf031. doi: 10.1093/eschf/xvaf031 (PMC13235861; doi:10.1093/eschf/xvaf031)
Supplement: xvaf031_Supplementary_Data [file xvaf031_supplementary_data.docx]

**Supplementary appendix**

This appendix has been provided by the authors to give readers additional information about their work.

Supplement to: Omar M, Jensen J, Ali M, et al. *Effects of Empagliflozin on Right Ventricular Function in Heart Failure with Reduced Ejection Fraction*

*A substudy from the Empire HF trial*

*Running title: Empire HF RV*

**Corresponding author**: Massar Omar, MD, PhD

**TABLE OF CONTENT**

Section A: List of investigators and steering committee1

Section B: Manufactured product Section2

Section C: Full list of inclusion and exclusion criteria3

Section D: Echocardiography4

Section E: Trial Profile based on RVFWS data6

Section F: Intention-to-treat population baseline characteristics 7

Section G: Correlations between RVFWS and other variables 9

Section H: Specified subgroups analyses for the lower RVFWS groups10

Section I: Section J: Subgroup analysis of TAPSE and RV S’…………………………………………….……………11

**Section A: List of investigators and steering committee**

**Investigators at Odense University Hospital, Odense, Denmark**

Principal investigator: Professor Jacob Eifer Møller, PhD, DSc

Sub-investigator: Massar Omar, PhD

**Investigators at Herlev and Gentofte University Hospital, Herlev, Denmark**

Principal investigator & sponsor: Professor Morten Schou, PhD

Sub-investigator: Jesper Jensen, PhD

**Steering committee:**

Lars Køber MD (chair), Jacob Eifer Møller MD, Morten Schou MD, Massar Omar MD, Jesper Jensen MD, Caroline Kistorp MD, Mikael Kjær Poulsen MD, Christian Tuxen MD, Ida Gustafsson MD, Finn Gustafsson MD, Emil Fosbøl MD, Niels Eske Bruun MD, Lars Videbæk MD.

**Section B: Manufactured product & Safety Variables**

**Primary Object**

Adult male and female patients with chronic heart failure and reduced ejection fraction.

**Manufactured product, doses and mode of administration**

To the existing standard of care treatment for heart failure, empagliflozin 10 mg or a matching placebo was administered orally once daily for 12 weeks period. The manufacture of empagliflozin was not involved nor funded the study.

**Section C: Full list of inclusion and exclusion criteria**

| **Inclusion Criteria** | **Exclusion Criteria** |
| --- | --- |
| Optimal HF therapy in accordance with European and national guidelines | CRT-D/-P implanted < 90 days |
| LVEF ≤ 0.40 | Uncorrected severe valvular heart disease |
| Estimated GFR > 30 ml/min/1.73 m^2^ | Non-compliance |
| BMI < 45 kg/m^2^ | Use of metalozone |
| NYHA functional class I-III | NYHA functional class IV |
| Age > 18 years | Age > 85 years |
|  | Dementia |
|  | Hospitalisation for HF < 30 days |
| If T2D – optimal treatment in accordance with European and national guidelines | Hospitalisation for hypoglycaemia < 12 months |
| If T2D – stable doses of antidiabetic treatment for 30 days | Known sustained ventricular tachycardia |
| If T2D – HbA1c 6.5–10.0% (48–83 mmol/mol) | Symptomatic hypotension and systolic blood pressure < 95 mmHg |
|  | Unable to perform an exercise test |
|  | Immobilization |
|  | Pregnancy |
|  | Participation in other medical trials |
|  | Previous intolerance of empagliflozin or excipients |

**Section D: Echocardiography**

**Echocardiography**Comprehensive transthoracic echocardiography (TTE) was performed by an experienced sonographer on a Vivid e9 ultrasound system (General Electric, Horten, Norway) and stored digitally for offline analysis. Images were analyzed blinded to treatment allocation in a random order. In addition, to maximize blinding, images obtained at Herlev and Gentofte University Hospital were analyzed at Odense University Hospital and vice versa. For two-dimensional (2D) and Doppler images, three consecutive beats for patients in sinus rhythm and multiple beats for patients with atrial fibrillation were measured and averaged. To ensure standardization of measurements, inter- and intra-observer reproducibility for LV parameters were assessed in 10 patients by calculating the intraclass correlation coefficient (ICC) at two different time points. ICC ranged from 0 to 1, and reliable values were considered to be > 0.6.

RVFWS was measured with Q-analysis from the apical four-chamber view, measured from the basal septum to the lateral RV wall, and from the apex to the base of the right ventricle (figure 1). Endocardial borders were traced, and speckles were tracked throughout the cardiac cycle from three standard apical views. TAPSE was measured in the A4C-view using the M-mode cursor over the lateral tricuspid annulus and measuring a straight line from top to bottom of the wave, which calculates the systolic displacement of the tricuspid annulus. The tricuspid peak gradient is measured where the curve is most legible with Doppler images over the tricuspid valve in the A4C-view. All measurements were standardized to body surface area (BSA). For two-dimensional and Doppler images, three consecutive beats for patients in sinus rhythm and multiple beats for patients with atrial fibrillation were measured and averaged.


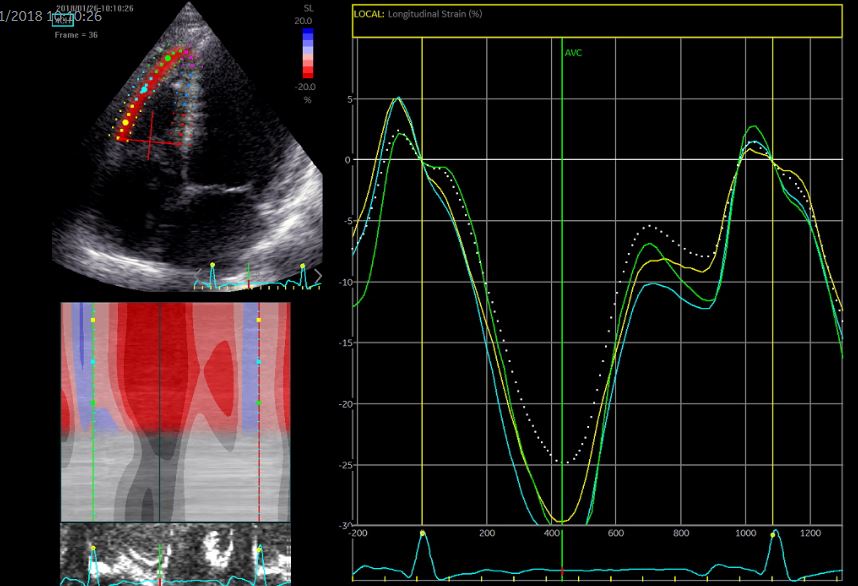


Figure 1: Right ventricle free wall strain measured by transthoracic echocardiography

**Section E: Trial Profile based on RVFWS data**

**486** Excluded

**305** Did not meet criteria

**131** Declined to participate

**50** Other reasons

**21** excluded

**12** Did not meet criteria

**8** Declined to participate

**1** Other reasons

**95** Assigned to placebo

**95** Received study drug

**95** Assigned to empagliflozin

**95** Received study drug

**1** Lost to follow-up

**0** Discontinued empagliflozin

**3** Lost to follow-up

**1** Discontinued placebo (no reason given)

**94** **C**ompleted the study

**82** included with available RVFWS data

**92** **C**ompleted the study

**78** included with available RVFWS data

**190** Randomized

**Follow-up**

**Analysis**

**697** Patients assessed for eligibility

**211** Patients screened

**Figure 1. Trial profile**

**Allocation**

**Enrolment**

| Figure 1: Overview of the trial profile and distribution in the study arms.  Abbreviations:; RVFWS, Right Ventricular Free Wall Strain; |
| --- |

**Section F: Intention-to-treat population baseline characteristics**

**Table 1.** Baseline Characteristics of intention-to-treat Population.

| **Table 1: Baseline Characteristics** | **Empagliflozin**  **n = 95** | **Placebo**  **n = 95** |
| --- | --- | --- |
|  |  |  |
| *Age, mean (SD), years* | 65 (10) | 63 (12) |
| Male, No. (%) | *79 (83)* | *83 (87)* |
| *Body mass index, median (IQR)* ^a^ | 29 (27–33) | 29 (26–33) |
| ***Heart failure characteristics*** |  |  |
| Duration of heart failure, mean (SD), months | 36 (12-69) | 27 (13-62) |
| Ischemic Heart failure cause, No. (%) | 48 (51) | 49 (52) |
| *Coronary Artery Bypass (CABG), No. (%)* | 15 (30) | 14 (26) |
| NYHA Class, No. (%) |  |  |
| *I* | 5 (5) | 7 (7) |
| *II* | 72 (76) | 77 (81) |
| *III* | 18 (19) | 11 (12) |
| Systolic blood pressure, mean (SD), mm Hg | 119 (18) | 121 (16) |
| Diastolic blood pressure, mean (SD), mm Hg | 69 (11) | 74 (12) |
| Mean pulse, mean (SD), beats/min | 70 (11) | 72 (13) |
| **Comorbidities, mean (SD), No. (%)** |  |  |
| Type 2 diabetes | 11 (12) | 13 (14) |
| Hypertension | 35 (37) | 41 (43) |
| Atrial Fibrillation | 33 (35) | 33 (35) |
| Ischemic heart disease | 50 (53) | 53 (56) |
| Chronic kidney disease^b^ | 13 (14) | 12 (13) |
| **Medications, mean (SD), No. (%)** |  |  |
| *ACE inhibitors/ARBs* | 58 (61) | 65 (68) |
| *Sacubitril–valsartan* | 31 (33) | 27 (28) |
| β blockers | 90 (95) | 89 (94) |
| Mineralocorticoid-receptor antagonist | 62 (65) | 63 (66) |
| Diuretics^c^ | 63 (66) | 62 (65) |
| **Laboratory variables** |  |  |
| NT-proBNP, median (IQR), ng/l | 582 (303-1020) | 605 (309-1080) |
| *In sinus rhythm* | 414 (276-689) | 473 (244-793) |
| *In atrial fibrillation* | 1050 (596-1820) | 1020 (581-1490) |
| Estimated glomerular filtration rate, median (IQR), mL/min/1.73m^2^ | 73 (57-89) | 74 (60-90) |
| Haematocrit, median (IQR), % | 42 (4) | 41 (4) |
| **Echo** |  |  |
| ***Left Ventricle*** |  |  |
| LV End Diastolic Volume, mean (SD), mL | 167 (79) | 159 (60) |
| LV End Systolic Volume, mean (SD), mL | 112 (66) | 104 (48) |
| LV Mass Index, mean (SD), g/m^2^ | 130 (64) | 128 (46) |
| LVEF, mean (SD), % | 37 (10 | 36 (10) |
| LV Global longitudinal strain, mean (SD), % | 11 (4) | 11 (3) |
| LA index, mean (SD), cm^2^/m^2^ | 41.4 (19.6) | 36.4 (12.9) |
| ***Right Ventricle*** |  |  |
| RV Free Wall Strain, mean (SD), % | 16.4 (6.3) | 16.9 (5.9) |
| TAPSE, mean (SD), cm | 2.0 (0.6) | 2.1 (0.6) |
| RV S' Free Wall, mean (SD), cm/s | 10.7 (3.0) | 10.9 (3.0) |
| TR maxPG, mean (SD), mmHg | 21.2 (12.3) | 20.0 (9.3) |

*Legend: Baseline characteristics of the empagliflozin and placebo group in patients with heart failure and reduced ejection fraction with available RVFWS measurements.*

*Abbreviations: NYHA, New York Heart Association; ACE inhibitors, Angiotensin-Converting Enzyme inhibitors; ARB, Angiotensin-II Receptor Blockers; NT-proBNP, N-terminal pro-B-type natriuretic peptides; LV, Left ventricle, LA, left atrium; RV, Right Ventricle; TAPSE, Tricuspid Annular Plane Systolic Excursion; TR maxPG, Tricuspid Regurgitation Peak Gradient; RV S', Right Ventricular Systolic Velocity.*

*SD, Standard Deviation; IQR, interquartile range [25% and 75% percentiles).*

** Diuretics includes Loop diuretics or Thiazide.*

**Section G: Correlations between RVFWS and other variables**

| **Table 2: Correlations of the Change in Right Ventricular Free Wall Strain** | | | | | | | | | | |
| --- | --- | --- | --- | --- | --- | --- | --- | --- | --- | --- |
|  |  |  |  |  |  |  |  |  |  |  |
|  | **Right ventricular Free Wall Strain** | | | | | **Right ventricular Free Wall Strain, lowest tertile (RVFWS ≤ 13 %)** | | | | |
|  |  |  |  |  | **Between group difference** |  |  |  |  | **Between group difference** |
|  | **Empagliflozin** | | **Placebo** | |  | **Empagliflozin** | | **Placebo** | |  |
|  | **R** | **p-value** | **R** | **p-value** | **p-value** | **R** | **p-value** | **R** | **p-value** | **p-value** |
| **Cardiac changes** |  |  |  |  |  |  |  |  |  |  |
| TAPSE | 0.00 | 0.99 | 0.07 | 0.59 | 0.64 | 0.19 | 0.38 | 0.16 | 0.49 | 0.93 |
| RV S' | -0.20 | 0.12 | -0.01 | 0.96 | 0.40 | -0.11 | 0.66 | -0.08 | 0.74 | 0.94 |
| TR _max_PG | -0.15 | -0.16 | -0.19 | 0.13 | 0.67 | -0.03 | 0.89 | -0.15 | 0.52 | 0.69 |
| LVEF | -0.05 | 0.68 | -0.01 | 0.95 | 0.86 | -0.04 | 0.85 | 0.03 | 0.91 | 0.82 |
| LA index | 0.02 | 0.91 | -0.12 | 0.34 | 0.34 | 0.18 | 0.42 | 0.05 | 0.82 | 0.77 |
| LVEDV | 0.03 | 0.82 | -0.12 | 0.31 | 0.31 | 0.40 | 0.06 | 0.07 | 0.75 | 0.32 |
| LVESV | 0.06 | 0.63 | -0.10 | 0.40 | 0.32 | 0.32 | 0.14 | 0.01 | 0.96 | 0.33 |
| Systolic blood pressure | -0.03 | 0.82 | -0.03 | 0.82 | 0.72 | 0.29 | 0.17 | -0.14 | 0.55 | 0.16 |
| Diastolic blood pressure | 0.10 | 0.41 | -0.09 | 0.46 | 0.27 | 0.41 | 0.06 | -0.04 | 0.85 | 0.14 |
| NT-proBNP | -0.01 | 0.95 | -0.10 | 0.42 | 0.48 | 0.09 | 0.68 | -0.20 | 0.40 | 0.25 |
| **Cardiac changes,** |  |  |  |  |  |  |  |  |  |  |
| B-Hematocrit | 0.01 | 0.92 | -0.03 | 0.79 | 0.77 | 0.04 | 0.86 | -0.21 | 0.35 | 0.40 |
| Plasma Volume | 0.02 | 0.90 | 0.01 | 0.93 | 0.99 | -0.08 | 0.71 | 0.15 | 0.53 | 0.43 |
| Weight | 0.06 | 0.61 | -0.03 | 0.78 | 0.61 | 0.10 | 0.64 | -0.13 | 0.58 | 0.42 |
| BMI | 0.06 | 0.65 | -0.05 | 0.70 | 0.55 | 0.13 | 0.55 | -0.16 | 0.49 | 0.32 |
| NYHA | -0.22 | 0.07 | -0.07 | 0.59 | 0.49 | 0.27 | 0.23 | 0.03 | 0.91 | 0.46 |

Legend: *Correlations of the change in right ventricular free wall strain with the change in other variables at 12 weeks in the intention-to-treat HFrEF population. P-values indicates significance (if p<0.05) in correlations between groups.
Abbreviations: RVFWS, Right Ventricular Free Wall Strain; TAPSE, Tricuspid Annular Plane Systolic Excursion; RV S', Right Ventricular Systolic Velocity; TR _max_PG, Tricuspid Regurgitation Peak Gradient; LVEF, Left Ventricular Ejection Fraction; LA index, Left Atrial Index; LVEDV, Left Ventricle End Diastolic Volume: LVESV, Left Ventricle End Systolic Volume; NT-proBNP, N-terminal pro–B-type natriuretic peptide; BMI, Body Mass Index; NYHA, New York Heart Association.*

**Correlations of the Change in RVFWS with the Change in LV reverse remodelling at 12 Weeks**

|  | **Empagliflozin, 10 mg/d** | | **Placebo** | | **Between-group difference** |
| --- | --- | --- | --- | --- | --- |
|  | ***R*** | ***P* value** | ***R*** | ***P* value** | ***P* value** |
| **Cardiac measurements** |  |  |  |  |  |
| LVESV (mL) | −0.10 | 0.68 | −0.24 | 0.29 | 0.48 |
| LVEDV (mL) | 0.40 | 0.06 | 0.07 | 0.74 | 0.35 |
| LVM (g/m^2^) | 0.17 | 0.44 | −0.15 | 0.51 | 0.31 |
| LAVI (ml/m^2^) | 0.18 | 0.42 | 0.05 | 0.82 | 0.79 |
| SBP (mmHg) | 0.29 | 0.19 | −0.14 | 0.55 | 0.18 |

Pearson’s correlation between the change in RVFWS and LV reverse remodelling. No significant correlations were observed between the change in RVFWS and LV reverse remodeling parameters.

LVESV, left ventricular end-systolic volume; LVEDV, left ventricular end-diastolic volume; LVM, left ventricular mas; LAVi, left atrial volume index; SBP, systolic blood pressure.

**Section H: Specified subgroups analyses for the lower RVFWS groups**

| **Figure 2:** Differences in characteristics between the empagliflozin and placebo groups in lowest tertile of RVFWS at baseline    Legend: Treatment Differences in the Primary Endpoint [RVFWS) in patients with ≥- 13.0 % RVFWS at baseline according to explorative Subgroups.  Forest plots are shown with number of patients in the subgroups at baseline. X-axis indicate change in RVFWS in absolute percentage; p values represents subgroups' comparison corresponding to test for interaction.  Abbreviations: Afib, atrial fibrillation; eGFR estimated glomerular filtration; HF, heart failure; NT-proBNP, N-terminal pro–B-type natriuretic peptide. |
| --- |

**Section I: Subgroup analysis of TAPSE and RV S’**

| **Table 3: Efficacy Measures n=160** |  |  |  |  |  |  |  |  |  |  |  |  |  |  |  |
| --- | --- | --- | --- | --- | --- | --- | --- | --- | --- | --- | --- | --- | --- | --- | --- |
|  | **Empagliflozin** | | | | | | **Placebo** | | | | | | **Between group** | | |
|  | **Baseline** | | **Follow-Up** | | **Mean change (SD)^a^** | | **Baseline** | | **Follow-Up** | | **Mean change (SD)^a^** | | **Treatment effect**  **[95% CI]^b^** | | **p-value** |
| **TAPSE, mean [SD], cm** |  |  |  |  |  |  |  |  |  |  |  |  |  |  |  |
| Entire cohort | 2.0 | [0.6] | 2.0 | [0.6] | 0.04 | [0.4] | 2.1 | [0.6] | 2.1 | [0.6] | 0.01 | [0.4] | - 0.05 | [-0.18 to 0.07] | 0.378 |
| Lower RVFWS tertile | 1.8 | [0.5] | 1.7 | [0.5] | - 0.14 | [0.4] | 1.8 | [0.5] | 1.7 | [0.5] | - 0.1 | [0.4] | -0.04 | [-0.21 to 0.29] | 0,758 |
| Middle RVFWS tertile | 2.0 | [0.5] | 2.0 | [0.5] | -0.01 | [0.4] | 2.2 | [0.5] | 2.2 | [0.4] | 0.0 | [0.5] | -0.01 | [-0.22 to 0.21] | 0.960 |
| Upper RVFWS tertile | 2.4 | [0.5] | 2.3 | [0.5] | -0.08 | [0.4] | 2.5 | [0.5] | 2.5 | [0.5] | 0.0 | [0.3] | - 0.16 | [-0.35 to 0.03] | 0.106 |
| **RV S', mean [SD], m/s** |  |  |  |  |  |  |  |  |  |  |  |  |  |  |  |
| Entire cohort | 0.11 | [0.03] | 0.10 | [0.03] | - 0.01 | [0.02] | 0.11 | [0.03] | 0.11 | [0.03] | 0.00 | [0.03] | -0.006 | [-0.014 to 0.002] | 0.141 |
| Lower RVFWS tertile | 0.09 | [0.03] | 0.09 | [0.02] | -0.01 | [0.02] | 0.10 | [0.03] | 0.09 | [0.02] | -0.02 | [0.03] | -0.001 | [-0.013 to 0.105] | 0.794 |
| Middle RVFWS tertile | 0.12 | [0.03] | 0.11 | [0.03] | - 0.01 | [0.03] | 0.11 | [0.03] | 0.09 | [0.03] | 0.00 | [0.023 | - 0.009 | [-0.025 to 0.006] | 0.227 |
| Upper RVFWS tertile | 0.11 | [0.02] | 0.11 | [0.02] | 0.00 | [0.02] | 0.12 | [0.03] | 0.12 | [0.024] | 0.01 | [0.02] | -0.007 | [-0.019 to 0.005] | 0.272 |

Legend*: Baseline and follow-up values for RV echocardiographic efficacy measures in HFrEF patients receiving either Empagliflozin (10 mg daily) or placebo for 12 weeks in subgroups stratified by RVFWS at baseline. Treatment effect of empagliflozin compared to placebo with their respective p-value. The population with available RVFWS has been sub-grouped in tertiles and is presented by these in the table.*
